# Thermodynamic and Transport Properties of Alkanes (C_*n*_H_2*n*+2_; *n* = 5–8)

**DOI:** 10.1002/open.202500254

**Published:** 2026-04-02

**Authors:** Deependra Awasthi, Narayan Gautam, Shyam P. Khanal, Rajendra Prasad Koirala, Narayan P. Adhikari

**Affiliations:** ^1^ Central Department of Physics Tribhuvan University Kathmandu Nepal

**Keywords:** free energy, shear viscosity, solvation, thermodynamic, transportation

## Abstract

In the present work, we have utilized “alchemical” approaches to study the thermodynamic properties of four alkane molecules: pentane, hexane, heptane, and octane in water. We have used thermodynamic integration (TI) and free energy perturbation (FEP) based methods: TI, TI‐cubic, Bennett acceptance ration (BAR), and Multistate Bennett acceptance ratio (MBAR)to estimate the solvation free energy of alkane molecules in water at 300 K for 21 distinct coupling parameter values (*λ*). For each alkane molecule, the estimated values of solvation free energy using the different methods are in close agreement, which ensures the reliability of our study. Convergence of the calculation has also been examined through time series plots in both forward and reverse directions. Our study shows that the solvation free energy increases with increasing size of the alkane molecules, which is also supported by estimation of solvent accessible surface area (SASA). The self diffusion coefficients of both solutes and solvent molecules; and shear viscosity of the systems have been estimated at 293 and 300 K temperatures. The self diffusion coefficient of the alkane molecules decreases with increase in size and shear viscosity increases with chain length as expected. The estimated values of diffusion and shear viscosity are in close agreement with the previously reported values.

## Graphical abstract

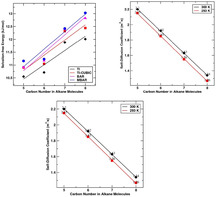

## 1 Introduction

Alkanes, the simplest family of hydrocarbons compound, are saturated compounds with the general formula C_*n*_H_2*n*+2_, characterized by single covalent bond [1, 2]. Their low reactivity, high boiling points, and nonpolar nature make them versatile in industrial and natural contexts [3]. The boiling temperature of alkanes rises as their molecular weight increases. For the corresponding molecular weights of 72.15, 86.18, 100.20, and 114.23 g/mol, the boiling temperatures of pentane (C_5_H_12_), hexane (C_6_H_14_), heptane (C_7_H_16_), and octane (C_8_H_18_) are 97.0°F, 156.0°F, 209.1°F, and 258.1°F, respectively [4, 5].

Because of the covalent bonds between carbon and hydrogen as well as the negligible variation in the electronegativities of carbon and hydrogen, alkanes are typically nonpolar molecules [6, 7]. The hydrophobic characteristic of alkanes is demonstrated by their nonpolarity, which prevents them from being drawn to polar molecules like water [8]. Alkane molecules with abundant oxygen (O_2_) easily burn to produce carbon dioxide, water, and energy [9, 10]. The energy thus generated can be applied to several aspects of daily life.

Alkanes are basically present in crude oil, natural gas, and biomass and are used as fuels, solvents, and chemical intermediates. Their weak van der Waals (vdW) forces and hydrophobic properties govern their behavior in polar and nonpolar solvents, influencing their applications in energy storage and biochemistry [11, 12]. Alkanes also serve as building blocks for biomolecules like fatty acids, which are integral to cellular processes and energy metabolism [13]. Individual alkane, exhibits unique physical and chemical properties that determine their specific industrial applications [14]. Pentane is used in foam production and hydrogen reforming; hexane is a key solvent in oil extraction and adhesives [15]; heptane aids in fuel octane rating and nonpolar solvent applications; and octane is applicable in high performance gasoline formulation. While these compounds are indispensable in various sectors, their handling requires strict safety measures due to their flammability and health hazards [16].

The thermodynamic behavior of alkanes, particularly their free energy, depends on factors such as molecular size, structure, temperature, and pressure. Larger alkanes generally exhibit higher free energy due to their increased molecular complexity and reduced conformational entropy [17]. This relationship, expressed through Gibbs free energy (Δ*G *= Δ*H *− *T*Δ*S*), underscores the influence of molecular structure on their stability and reactivity [18]. Research into these properties not only enhances understanding of alkanes in chemical systems but also informs the development of applications in fuel efficiency, drug discovery, and materials science [19].

Designing optimal fuels, lubricants, and chemical feedstocks of alkanes molecules requires a detailed understanding of their thermodynamic and kinetic properties [20, 21]. Research on these properties, especially the role of free energy, is crucial to advance energy storage systems, improve combustion processes, and develop environmentally friendly solutions [22]. This work delves into the solvation free energies of some alkanes (pentane, hexane, heptane, and octane) to explore their solubility, transportation and molecular interactions in aqueous environments. These insights have broad applications, from energy production to materials science, highlighting the importance of such investigations in both fundamental and applied research [23, 24].

Overall, the study “thermodynamic and transport properties” of alkane molecules offers valuable insights into their role in energy systems, biochemistry, and industrial processes. By exploring their solubility, molecular interactions, and structural influences, cleaner energy solutions can be developed and are used to improve material properties, and discover innovative applications [25]. The thermodynamic and transport properties of an Amoxicillin, peptides of some amino acids like Valine, Cystine, Leucine, and Isoleucine in aqueous enviroment have been already studied using molecular dynamics (MD) study [26, 27, 28]. Moreover, the transport properties of the first four alkanes: methane, ethane, propane, and butane have already been examined [29]; however, computational studies on the thermodynamic and transport properties of higher alkanes like pentane, hexane, heptane, and octane have yet to be studied. In the present work, MD study has been performed to understand transport and thermodynamic properties of the alkane molecules.

## 2 Materials and Methods

### 2.1 Theoretical Background

MD simulation, a computational method, is deterministic approach in which macroscopic properties can be studied from the analysis of trajectory followed by particles using statistical mechanics [30, 31]. The technique simulates particle's motion to explore physical and chemical phenomena at the atomic level; and provides more insight into macromolecular structure–function relationships, transport properties, and thermodynamic characteristics, offering a cost‐effective and efficient alternative to experimental methods. Based on such method, we have performed classical MD simulations to study the thermodynamic property like solvation free energy and transport properties including diffusion coefficient and shear viscosity of some alkane molecules: pentane, hexane, heptane, and octane in aqueous medium.

#### 2.1.1 Free Energy of Solvation

Free energy calculation is a fundamental technique for understanding many biophysical and chemical processes, such as protein folding, solvation, and binding thermodynamics [32, 33]. Free energy, a state function, help to understand quantitatively the system's usable energy for work [34, 35]. The difference in free energy between two thermodynamic states, initial state “A” and final state “B”, can be estimated using free energy perturbation (FEP) approach as [36]

(1) $$\Delta F_{\mathrm{AB}}=-k_{B}T\ln\langle {\exp\{-\beta (U_{B}-U_{A})\}\rangle }_{A}$$

where *β* = 1/*k*_B_ *T*. Here, the initial and final states are represented by potential energy *U*_A_ and *U*_B_ respectively; and ${\left\langle \ldots \right\rangle }_{A}$ represents averaging over state A, enabling incremental computation of Δ*F*_AB_ by simulating small changes in potential energy. Moreover, thermodynamic integration (TI) approach calculates the free energy difference Δ*F*_AB_ by integrating the ensemble‐averaged derivative of the potential energy (⟨∂U/∂λ⟩) along a coupling parameter *λ* (with values 0 ≤ *λ *≤ 1) that interpolates between states A and B [36]

(2) $$\Delta F_{\mathrm{AB}}=\int_{0}^{1}{\left\langle \frac{\partial U}{\partial \lambda }\right\rangle }_{\lambda }d\lambda$$

The values 0, 1, and other of the parameter *λ* represent the initial, final, and nonphysical intermediate states, respectively. This approach provides a robust framework for determining free energy differences by generating a thermodynamic path and sampling intermediate states. Both methods leverage statistical mechanics and computational simulations to calculate free energy differences, offering critical insights into molecular systems energetics.

#### 2.1.2 Diffusion Coefficient

Diffusion is the process where particles move from a region of high concentration to low concentration due to random motion [37]. Diffusion plays a crucial role in various physiological processes, such as biomolecule dynamics, nutrient transport, and drug delivery [38]. This transport property is measured by the diffusion coefficient, which quantifies the rate of movement of particles through a medium and depends on factors such as temperature and particle size [31, 39]. Using the mean squared displacement (MSD) ⟨*r* ^2^(*t*)⟩, the self diffusion coefficient can be calculated via Einstein's relation [40]

(3) $$D=\lim_{t\to \infty }\frac{\left\langle {\left[r_{\alpha }(t+t_{0})-r_{\alpha }(t_{0})\right]}^{2}\right\rangle }{6t}$$

where *α* indicates particle type and ⟨···⟩ represents an ensemble average.

#### 2.1.3 Shear Viscosity

Shear viscosity, also known as apparent viscosity, quantifies the relationship between shear stress and shear rate in a fluid. For Newtonian fluids, this relationship remains constant and is governed by Newton's Law of Viscosity [41]. Shear viscosity can be derived from an equilibrium simulation using the Einstein relation as [42]

(4) $$\eta =\frac{1}{2}\frac{V}{k_{B}T}\lim_{t\to \infty }\frac{d}{dt}{\left\langle {\left(\int_{t_{0}}^{t_{0}+t}P_{xz}(t\prime )\;dt\prime \right)}^{2}\right\rangle }_{t_{0}}$$

In Equation (4), the parameters *η*, *V*, *T*, *k*_B_, *P*_*xz*_(*t*′), and ⟨…⟩ represents the shear viscosity, volume, temperature, Boltzmann constant, pressure component (stress), and average over time origins, respectively. This method converges slowly and is computationally demanding due to the noise introduced in the off diagonal pressure elements, particularly when short‐range cutoffs are applied for electrostatics. These factors significantly impact the calculated viscosity, sometimes increasing it by an order of magnitude [42, 43].

### 2.2 Computational Details

We used GROningen MAchine for Chemical Simulations (GROMACS) software package [44] for all simulations. To perform MD simulations, we considered following four systems with binary mixtures of: one pentane molecule in 598 water molecules (System‐I), one hexane molecule in 599 water molecules (System‐II), one heptane molecule in 681 water molecules (System‐III), and one octane molecule in 787 water molecules (System‐IV). Visualization tools such as VMD and PyMOL were used during simulations by enabling three‐dimensional molecular visualization, structural analysis, and data arrangement, with VMD excelling in dynamic rendering and PyMOL in interactive exploration and high‐quality graphics [45, 46]. All the solute molecules were modeled using Optimized Potential for Liquid Simulations‐All Atom (OPLS‐AA) forcefield; and Transferable Intermolecular Potential with 3‐Points (TIP3P) water model used as solvent [47, 48]. All simulations carried out in cubic box at 1 atm pressure under periodic boundary conditions to handle surface effect [31, 49].

#### 2.2.1 Estimation of Solvation Free Energy

We performed MD simulations to estimate the solvation free energy of aforementioned alkane molecules in water at 300 K temperature. In order to estimate free energy, we introduced the nonphysical intermediate states through the manupulation of only nonbonded vdW and Coulomb interactions [50]. The 21 different values of coupling parameter (*λ*) used to manupulate Coulomb and vdW interactions are [26, 27]

*λ*_Coulomb _= 0.00, 0.10, 0.20, 0.30, 0.40, 0.50, 0.60, 0.70, 0.80, 0.90, 1.0, 1.00, 1.00, 1.00, 1.00, 1.00, 1.00, 1.00, 1.00, 1.00, and 1.00 for Coulomb interaction and *λ*_vdW _= 0.00, 0.00, 0.00, 0.00, 0.00, 0.00, 0.00, 0.00, 0.00, 0.00,0.00, 0.10, 0.20, 0.30, 0.40, 0.50, 0.60, 0.70, 0.80, 0.90, and 1.00 for vdW interaction.

The states *λ *= 0 and *λ *= 1 represent the solute and solvent molecules which are fully coupled and decoupled, respectively.

After system set up, energy minimization run was carried taking each system to remove vdW bad contact using steepest descent method [44]. After energy minimization of each system, equilibration runs were carried out for 5 ns with time step of 2 fs under NVT ensemble at first followed by NPT ensemble to bring the system in thermodynamic equilibrium [51]. Initial velocity for each particle was assigned using the concept of Maxwell‐Boltzmann distribution. All bonds were constrained by using LINCS algorithms and equations of motion solved through Langevin dynamics [52, 53]. In addition, Parrinello‐Rahman barostat with coupling time 1 ps and value of isothermal compressibility 4.5 × 10^−5^ bar were used in each equilibration run [54]. Finally, production runs were performed taking each equilibrated system for 5 ns with time step 2 fs using Langevin dynamics. We used 1.2 nm cutoff parameter for both LJ and Coulomb interactions; and we accounted long range Coulomb interaction by considering Particle Mesh Ewald (PME) during each equilibration as well as production run [53].

#### 2.2.2 Transport Properties

Furthermore, to study the transport properties, different molecular systems were set up and MD simulations were carried out taking above four molecules in cubic simulation box. Energy minimization run was performed for each system. Each of the system was then equilibrated at temperature 293 and 300 K under isothermal–isobaric (NPT) ensemble with time step of 2 fs for 500 ns time using Leap‐frog algorithms [53]. During each equilibration run, the long range Coulomb interaction was handled using Particle Mesh Ewald (PME) method; and 1 nm cut off length was chosen for both Coulomb and LJ interactions as discussed in Section 2.2.1. Maxwell‐Boltzmann distribution and LINCS algorithm were used to assign initial velocity for each particle and to constrain all bonds, respectively [52]. To control the temperature and pressure, velocity rescaling thermostat was used with coupling time of 0.01 ps and Berendsen barostat with coupling time 0.8 ps, respectively [55, 56]. After each equilibration run, density of the molecular system was estimated and was compared with experimental results, which gave the close agreement. Finally, production runs were performed considering each of the equilibrated system at both the temperatures: 293 and 300 K under NVT condition for 1000 ns with time step of 2 fs taking same parameters used during equilibration run.

## 3 Results and Discussion

This study provides the insights into thermodynamic and transport properties focusing on solvation free energy, self and binary diffusion coefficients, and shear viscosity of alkane molecules (C_*n*_H_2*n*+2_; *n* = 5–8) in aqueous enviroment.

### 3.1 Free Energy of Solvation

**FIGURE 1:**
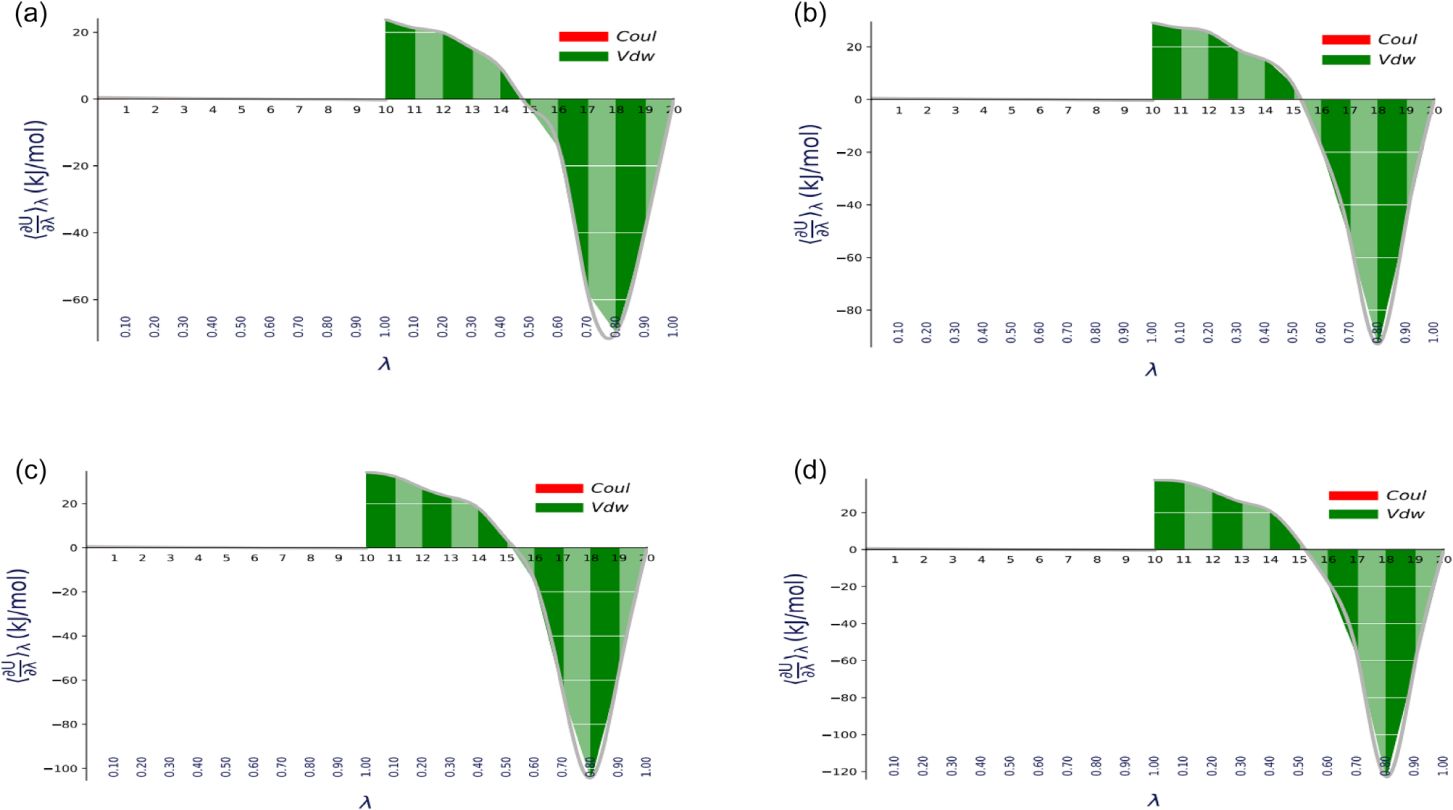
Variation of ${\left\langle \frac{\partial U}{\partial \lambda }\right\rangle }_{\lambda }$ with *λ* for (a) pentane, (b) hexane, (c) heptane, and (d) octane taking TIP3P water model as solvent at temperature 300 K.

The solvation free energy of the alkane molecules (C_*n*_H_2*n*+2_; *n* = 5–8) has been estimated using two TI based methods: TI and TI‐CUBIC that uses trapezoidal and cubic spline rules numerical integration, respectively; and FEP based Bennett acceptance (BAR) and Multistate Bennett acceptance (MBAR) methods. Python tool “alchemical‐analysis.py” was used to estimate the free energy difference between two thermodynamic states from the analysis of trajectory followed by particles [50, 57]. In order to overcome the problem of convergence during estimation of free energy difference, we introduced many nonphysical intermediate states between initial and final states using the concept of coupling parameter (*λ*) [58]. The states *λ *= 0, *λ *= 1, and other intermediate values between 0 and 1 represent initial, final, and nonphysical states, respectively. In this calculation, the initial state (*λ *= 0) represents the thermodynamic state in which solute and molecules are fully coupled through nonbonded interactions: vdW and Coulomb; and final state (*λ *= 1) represent the decoupled state, i.e., solute is free from solvent. TI based methods evaluate the solvation free energy between two states from the variation of ${\left\langle \frac{\partial U}{\partial \lambda }\right\rangle }_{\lambda }$ with coupling parameter *λ*. Figure 1a–d represent the plots between ${\left\langle \frac{\partial U}{\partial \lambda }\right\rangle }_{\lambda }$ and *λ* for different alkanes in TIP3P water at temperature 300 K.

In Figure 1a–d, red and green color (online) represent the contribution of Coulomb and vdW interactions to the solvation free energy of the alkane molecules, respectively. During our simulations, we first slowly turned off the Coulomb interction upto 11th states keeping *λ*_vdW_ = 1 ; and then gradually turrned off vdW. From Figure 1a–d, it is observed that only vdW interaction contribute to the solvation free energy and there is no significant contribution due to Coulomb interaction which is expected in alkane molecules. The estimated values of solvation free energy of different alkane molecules in water at 300 K using different methods are presented in Table 1.

**TABLE 1:** Estimated values of free energy of solvation (Δ*G*_solv_) in kJ/mol for alkane molecules in TIP3P water at 300 K taking individual contributions of vdW and Coulomb with total using TI, TI‐CUBIC, BAR, and MBAR methods.

Alkane molecules	Method	**Δ** ***G*** **_solv_** **in kJ/mol with**		
		vdW only	Coulomb only	Total
Pentane	TI	10.62 ± 0.15	−0.07 ± 0.00	10.55 ± 0.15
	TI‐CUBIC	10.97 ± 0.15	−0.06 ± 0.00	10.90 ± 0.15
	BAR	10.98 ± 0.11	−0.07 ± 0.00	10.91 ± 0.11
	MBAR	11.22 ± 0.14	−0.06 ± 0.00	11.16 ± 0.14
Hexane	TI	10.80 ± 0.15	−0.09 ± 0.00	10.71 ± 0.15
	TI‐CUBIC	11.14 ± 0.15	−0.09 ± 0.00	11.05 ± 0.15
	BAR	11.26 ± 0.11	−0.09 ± 0.00	11.17 ± 0.11
	MBAR	11.32 ± 0.14	−0.09 ± 0.00	11.23 ± 0.14
Heptane	TI	11.98 ± 0.11	−0.10 ± 0.00	11.88 ± 0.11
	TI‐CUBIC	12.43 ± 0.11	−0.10 ± 0.00	12.32 ± 0.11
	BAR	12.51 ± 0.09	−0.10 ± 0.00	12.40 ± 0.09
	MBAR	12.53 ± 0.11	−0.11 ± 0.00	12.42 ± 0.11
Octane	TI	12.13 ± 0.12	−0.12 ± 0.00	12.01 ± 0.12
	TI‐CUBIC	12.56 ± 0.11	−0.12 ± 0.00	12.44 ± 0.12
	BAR	12.94 ± 0.10	−0.12 ± 0.00	12.82 ± 0.10
	MBAR	13.15 ± 0.12	−0.12 ± 0.00	13.03 ± 0.12

**FIGURE 2:**
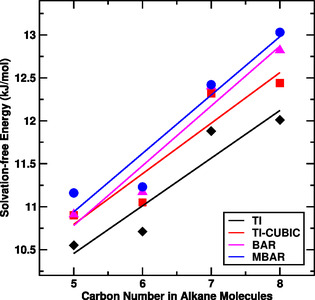
Solvation free energy as a function of carbon number in the alkane molecules at 300 K temperature.

The estimated values of solvation free energy, in Table 1, for each alkane molecule using TI, TI‐CUBIC, BAR, and MBAR are in closed agreement. This ensures the validation of reliability of our calculation. Solvation free energy of the alkane molecules increases on increasing number of carbon atoms in alkane. The results demonstrate stronger solute–solvent interactions due to increased surface area and hydrophobic effect with increase in size of alkane molecules as expected. We have also studied the variation of solvation free energy in water as a function of number of carbon atoms in the alkane molecules. Figure 2 represents the variation of solvation free energy as a function of size of alkane molecules.

**FIGURE 3:**
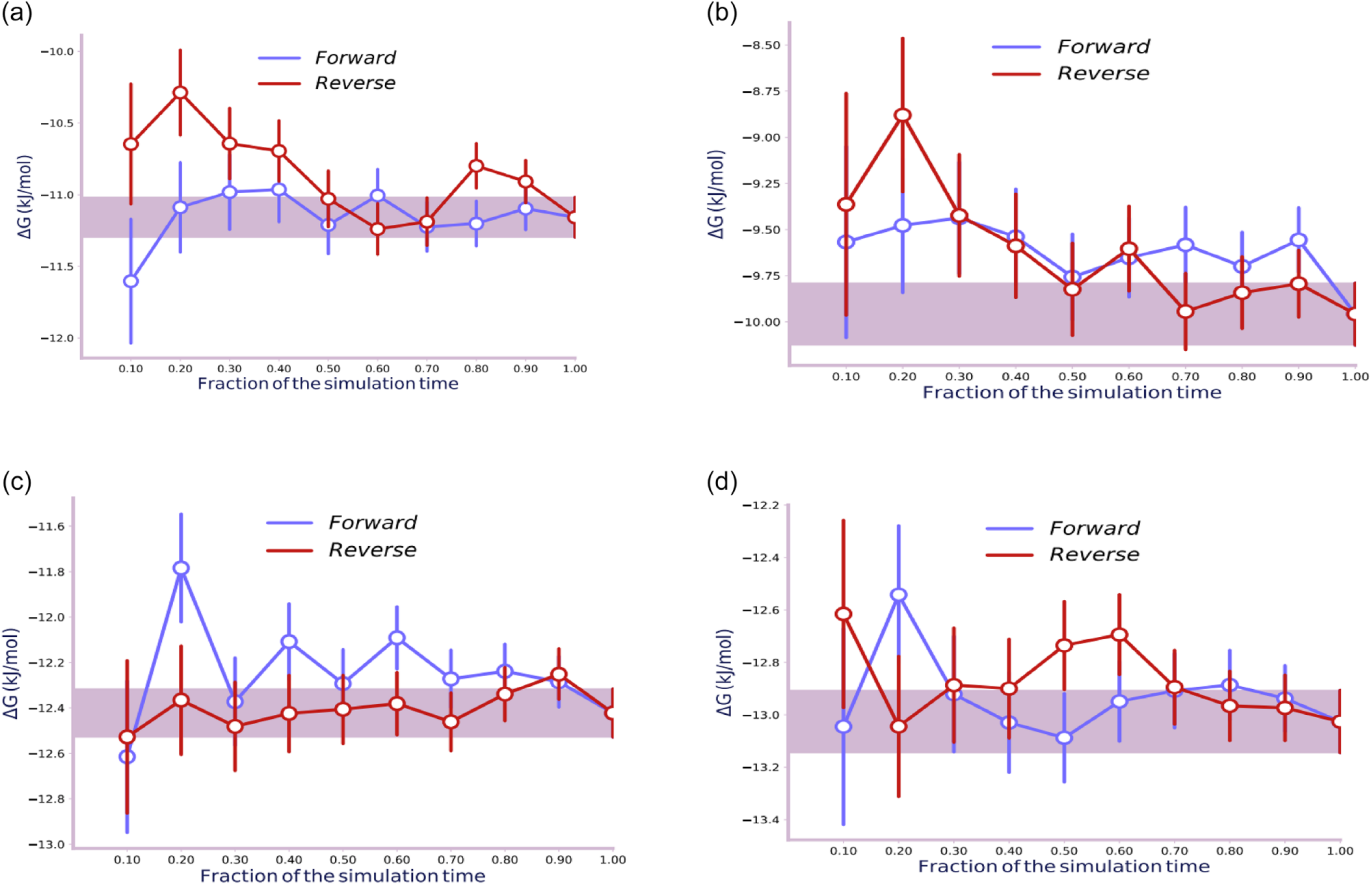
Variation of the estimated solvation free energy of the alkane molecules: (a) pentane, (b) hexane, (c) heptane, and (d) octane in TIP3P water over simulation time in both forward and reverse directions.

The solvation free energy estimated using various methods, in Figure 2, increases linearly with increasing number of carbon atoms in the alkane molecules. We have also examined the convergence of our calculation from the time series plots as shown in Figure 3a–d.

The time series plots demonstrate that the free energy calculations converge after a fraction of the simulation time, with the forward and reverse Δ*G* values closely agreeing after the initial hysteresis effect [50]. This confirms that the free energy estimates reach equilibrium, validating the reliability of the computed solvation free energy.

### 3.2 Solvent Accessible Surface Area (SASA)

**FIGURE 4:**
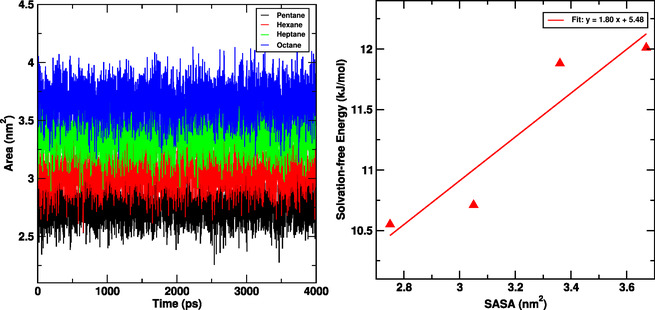
Time evolution of solvent accessible surface area (SASA) (left) and solvation free energy difference versus SASA values (right) of alkane molecules at 300 K.

Solvent accessible surface area (SASA) gives information on a interaction specific molecule with the aqueous environment. In Figure 4, the SASA of each molecule demonstrates a continual contact with water molecules and increases for larger molecules. This study demonstrates that the SASA has a substantial influence on the solvation free energy of alkane molecules in water. Larger molecules have higher SASA values and greater solvation free energies, indicating strong solute–solvent interactions, as shown in Figure 4. The solvation free energy can be estimated using the relation: ΔG=γ.SASA+C; where γ represents the surface tension parameter which gives the contribution to the nonpolar solvation free energy per unit surface area [59]. The positive correlation between SASA and solvation free energy is further validated by linear regression, confirming that molecular size and surface area are major factors in solvation behavior, with the graph in Figure 4 highlighting a clear correlation where an increase in SASA leads to a more solvation free energy, quantified by a slope of 1.80 kJ/mol per nm^2^, indicating a 1.80 kJ/mol increase in solvation free energy for every 1 nm^2^ increase in SASA.

In addition, we have also investigated the transport properties of the alkane molecules in water focusing on diffusion coefficient and shear viscosity.

### 3.3 Transport Properties

We have estimated the self diffusion coefficient of the solute molecules and water as well as their binary diffusion coefficient; and shear viscosity at 293 and 300 K temperatures.

#### 3.3.1 Diffusion Coefficients

The self diffusion coefficient of both solute and solvent have estimated using Einstein's relation 3. As self diffusion coefficient can be estimated from the slope of MSD versus time, we first plotted the variation of MSD as a function of time. For better statistics, we linearly fitted the plot taking begining portion of the trajectory for 5 ns. The estimated values of self diffusion coefficients at 293 and 300 K are presented in the Table 2.

**TABLE 2:** Simulated values of self‐diffusion coefficients of alkane molecules at 293 and 300 K temperature.

	**Self‐diffusion coefficient (** **×** **10** **^−9^** **m** **^2^** **/s) at temperature**						
Alkane molecules	293 K			300 K			
	Alkane		Water	Alkane		Water	
	Estimated	Ref. [60]	Estimated	Estimated	Ref. [60]	Estimated	Ref. [61]
Pentane	2.15 ± 0.01	2.24	5.00 ± 0.10	2.20 ± 0.01	2.54	5.67 ± 0.29	5.4
Hexane	1.85 ± 0.01	1.90	5.04 ± 0.22	1.92 ± 0.01	2.45	5.53 ± 0.01	
Heptane	1.55 ± 0.01	1.68	4.90 ± 0.08	1.63 ± 0.01	1.90	5.54 ± 0.15	
Octane	1.27 ± 0.01	1.46	5.06 ± 0.05	1.34 ± 0.01	1.63	5.51 ± 0.21	

**FIGURE 5:**
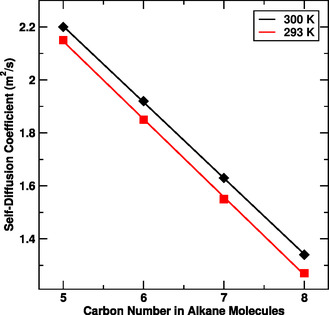
Self‐diffusion coefficient versus alkane molecules at different temperature.

From the Table 2, we have observed that the self diffusion coefficient of the alkane molecules decreases with increase in both size of the alkane as well as temperature as expected. The diffusion of smaller alkanes, such as pentane, hexane, and heptane, exhibited higher mobility compared to the larger alkane, octane, due to their shorter carbon chains and lower intermolecular forces. The diffusion coefficients at 293 K were reduced by ≈2%–3%, further highlighting the temperature dependence of diffusion. In addition, the self diffusion coefficient of water has been estimated and compared with previously reported value, and our estimated values are in close agreement. The experimentally reported value of self diffusion coefficient of water at 293 K temperature is 2.02 × 10^−9^ m^2^/s [62]. Our simulated values are higher than the experimentally reported value because we used TIP3p water model which overestimate diffusion coefficient [61]. We have also check variation of self diffusion coefficient of the alkane molecules with size. Figure 5 shows the variation of self diffusion coefficient of the alkane molecules with number of carbon atoms.

The study provides insight into the diffusive behavior of alkane molecules in aqueous environments, emphasizing the role of molecular size and thermal conditions in determining diffusion rates. The observed trend, where smaller alkanes diffuse more rapidly than larger ones, aligns with theoretical expectations and underscores the importance of molecular characteristics in transport properties.

#### 3.3.2 Shear Viscosity

Moreover, we used MD simulations to estimate shear viscosity of the alkane molecules: pentane, hexane, heptane, and octane in water at 293 and 300 K temperatures by using Einstein's relation in Equation (4) . By dividing the trajectory into multiple chunks and averaging the results, we have estimated the shear viscosity of each system. There is a close agreement between the estimated values and previously reported experimental data, shown in Table 3. The results highlighted the temperature dependence of viscosity, with values decreasing as the temperature increased, in line with experimental trends as shown in figure and table. This behavior is attributed to the reduced intermolecular forces at higher temperatures.

**TABLE 3:** Shear viscosity of alkane molecules at 293 and 300 K temperature.

Alkane molecules	**Shear viscosity (** **×** **10** **^−4^** **kg m** **^−1^** **s** **^−1^** **) at temperature**			
	293 K		300 K	
	Estimated	Ref. [63]	Estimated	Ref. [63]
Pentane	2.53 ± 0.01	2.70	2.31 ± 0.01	2.30
Hexane	3.06 ± 0.01	3.10	2.83 ± 0.01	2.93
Heptane	3.51 ± 0.01	3.91	3.20 ± 0.01	3.56
Octane	3.80 ± 0.01	4.22	3.52 ± 0.01	3.91

**FIGURE 6:**
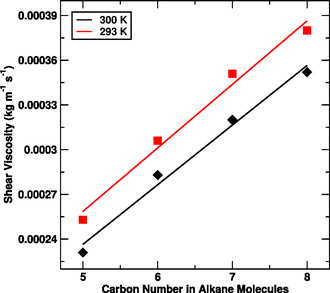
Shear viscosity versus carbon atoms in alkane molecules at 293 and 300 K temperatures.

From the Table 3, it is seen that the shear viscosity of the alkane molecules in water increases with size of alkane molecules. However, the shear viscosity decreases with rise in temperature in each of the system. We also have examined the nature of varying shear viscosity with number of carbon atoms. The result follows the linear trend as shown in Figure 6.

The shear viscosity depends on molecular size, with smaller molecules like pentane exhibiting lower viscosity due to weaker intermolecular interactions, while larger molecules like octane showed higher viscosity due to stronger vdW interactions and more entangled chains. These trends confirm the reliability of the simulations and demonstrate the utility of MD in studying temperature‐dependent viscosity. The insights gained are valuable for understanding the rheological properties of alkanes and can be applied to fields such as material science, chemical engineering, and petroleum industries, where knowledge of the flow behavior of hydrocarbons is essential.

## 4 Conclusions and Outlook

The thermodynamic and transport properties of some alkane molecules: pentane, hexane, heptane, and octane in an aqueous environment have been studied using MD. During system set up, OPLS‐AA force field and TIP3P water model were used. The solvation free energy of aforementioned alkane molecules in water have been estimated at 300 K temperature using thermodynamic and FEP based methods: TI, TI‐Cubic, BAR and MBAR. The value of solvation free energy increases with increase in size of the alkane molecules. Our results show that vdW interaction has the major contribution on solvation free energy of the alkane molecules in water. We have also explored solvent environment's impact on solvation‐free energy by calculating the SASA, which increases with the alkane chain length, further supporting to our observation that solvation free energy increses with the size of the alkane molecule.

We have also estimated the self diffusion coefficient of each solute and solvent molecules at 293 and 300 K temperatures using Einstein's relation. The self diffusion coefficient of alkane molecules decreases with increase in size as expected. The calculated values of the diffusion coefficient closely align with previously reported data, validating the accuracy of the simulations. Furthermore, shear viscosity of each system has also been calculated, offering further insights into the fluid properties. Overall, these findings provide a comprehensive understanding of the solvation and transport properties of alkane molecules in aqueous environments, highlighting the influence of molecular size and solvent interactions.

## Author Contributions

D.A. carried out the simulation work and contributed to the preparation of the manuscript draft, N.G. assisted D.A.; and S.P.K., R.P.K., and N.P.A. conceived the idea and finalized the manuscript. All authors read and approved the final manuscript.

## Funding

The authors have nothing to report.

## Conflicts of Interest

The authors declare no conflicts of interest.

## Data Availability

The data that support the findings of this study are available from the corresponding author upon reasonable request.
